# The revised Temperament and Character Inventory: normative data by sex and age from a Spanish normal randomized sample

**DOI:** 10.7717/peerj.1481

**Published:** 2015-12-22

**Authors:** Alfonso Gutierrez-Zotes, Javier Labad, Lourdes Martorell, Ana Gaviria, Carmen Bayón, Elisabet Vilella, C. Robert Cloninger

**Affiliations:** 1 Department of Psychiatry, Hospital Universitario Pere Mata, IISPV, Universitat Rovira i Virgili, CIBERSAM, Ctra. de l’Institut Pere Mata, s/n, Reus, Spain,; 2 Department of Psychiatry, Corporació Sanitària Parc Taulí, I3PT, UAB. Sabadell, Barcelona, Spain; 3 Hospital Universitario La Paz, Madrid, Spain; 4 Departament of Psychiatry, Washington University School of Medicine, St. Louis, USA

**Keywords:** Personality, Character, Temperament, TCI-R, TCI-140, Randomized sample, Inventory

## Abstract

**Objectives.** The psychometric properties regarding sex and age for the revised version of the Temperament and Character Inventory (TCI-R) and its derived short version, the Temperament and Character Inventory (TCI-140), were evaluated with a randomized sample from the community.

**Methods.** A randomized sample of 367 normal adult subjects from a Spanish municipality, who were representative of the general population based on sex and age, participated in the current study. Descriptive statistics and internal consistency according to *α* coefficient were obtained for all of the dimensions and facets. *T*-tests and univariate analyses of variance, followed by Bonferroni tests, were conducted to compare the distributions of the TCI-R dimension scores by age and sex.

**Results.** On both the TCI-R and TCI-140, women had higher scores for Harm Avoidance, Reward Dependence and Cooperativeness than men, whereas men had higher scores for Persistence. Age correlated negatively with Novelty Seeking, Reward Dependence and Cooperativeness and positively with Harm Avoidance and Self-transcendence. Young subjects between 18 and 35 years had higher scores than older subjects in NS and RD. Subjects between 51 and 77 years scored higher in both HA and ST. The alphas for the dimensions were between 0.74 and 0.87 for the TCI-R and between 0.63 and 0.83 for the TCI-140.

**Conclusion.** Results, which were obtained with a randomized sample, suggest that there are specific distributions of personality traits by sex and age. Overall, both the TCI-R and the abbreviated TCI-140 were reliable in the ‘good-to-excellent’ range. A strength of the current study is the representativeness of the sample.

## Introduction

The personality paradigm proposed by Cloninger and colleagues (Cloninger, 1987; Cloninger, Svrakic & Przybeck, 1993) provides a dimensional alternative for studying personality. It posits that there is a contribution from biological mechanisms and from learning through interactions in the development of an individual in his environment. As such, all personality dimensions are heritable yet character is greatly influenced by sociocultural factors (Josefsson et al., 2013a). Temperament and character are distinct domains of personality that interact as a dynamical non-linear system as emotional and rational processes are integrated throughout the lifespan (Josefsson et al., 2013a; Josefsson et al., 2013b; Cloninger, 2008). The revised Temperament and Character Inventory (TCI-R) is the third stage of development of a widely used multiscale personality inventory that began with the Tridimensional Personality Questionnaire (TPQ) and then the Temperament and Character Inventory (TCI).

All personality modules involve person by situation interactions that are regulated by a set of dynamical nonlinear systems, which allows human beings to be purposeful, flexible, and self-aware in their adaptation to life. Human personality is not adequately characterized as a set of linear traits because the components of personality are nonlinear in their functional effects and relationships with one another. As a result, personality is a complex expression of nonlinear interactions among a whole hierarchy of learning systems that have evolved and that develop over time as a complex adaptive process, as described in detail elsewhere (Cloninger, 2002; Turner et al., 2003; Cloninger, 2004; Cloninger, 2008; Cloninger & Kedia, 2011; Cloninger, 2015).

The revised Temperament and Character Inventory (Cloninger, Przybeck & Svrakic, 1999) is a self-administered dimensional questionnaire designed to evaluate the 7 basic dimensions of the Psychobiological Model of Personality (Table 1). Cloninger’s personality model includes 4 temperament and 3 character dimensions. The temperament dimensions are: Novelty seeking (NS; Exploratory excitability, Impulsiveness, Extravagance and Disorderliness) is defined as the tendency to respond impulsively to novel stimuli with active avoidance of frustration. It reflects the tendency to pursue reward and escape from punishment. Harm avoidance (HA; Anticipatory worry, Fear of uncertainty, Shyness with strangers and Fatigability) is the tendency to inhibit responses to aversive stimuli leading to avoidance of punishment and nonreward. Reward dependence (RD; Sentimentality, Openness to warm, Attachment and Dependence) is the tendency for positive attachment and response to signals of reward that maintain behavior. Persistence (PS; Eagerness of effort, Work hardened, Ambitious and Perfectionist) is the tendency to perseverance despite frustration and fatigue based on resistance to extinction of reinforced behavior. The character dimensions are as follows: Self-directedness (SD; Responsibility, Purposefulness, Resourcefulness, Self-acceptance and Enlightened second nature) refers to the executive ability of an individual to control, regulate, and adapt behavior to fit the situation in accordance to personal goals. Cooperativeness (CO; Social acceptance, Empathy, Helpfulness, Compassion, Pure-hearted conscience) accounts for individual differences in the acceptance of other people. (3) Self-transcendence (ST; Self-forgetful, Transpersonal identification and Spiritual acceptance) is viewed as the identification with everything conceived as essential and consequential parts of a unified whole (Cloninger, Svrakic & Przybeck, 1993; Cloninger et al., 1994). The TCI-140 represents a short form of the original TCI-R. It provides a score for the temperament (novelty seeking, harm avoidance, reward dependence, and persistence) and character (self-directedness, cooperativeness, and self-transcendence) dimension of the TCI-R, as well as separate scores for each facet.

**Table 1:** TCI-R descriptive, alpha, means comparison for sex and, correlations with age.

Facets and scales	*r* Age	Items	All (*n* = 367)		Females (*n* = 207)			Males (*n* = 160)				
			M (SD)	*α*	M (SD)	M (SD)^***^	*α*	M(SD)	M (SD)^***^	*α*	Cohen’s *d*	*p*
NS1.Exploratory excitability	−.38^**^	10	29.11 (6.05)	.53	28.89 (6.15)	2.88 (.61)	.54	29.39 (5.90)	2.93 (.59)	.52	−.08	.437
NS2.Impulsiveness	−.06	9	21.74 (5.48)	.56	21.32 (5.57)	2.36 (.61)	.59	22.28 (5.32)	2.47 (.59)	.52	−.17	.099
NS3.Extravagance	−.31^**^	9	24.99 (6.10)	.66	25.00 (6.54)	2.77 (.72)	.72	24.98 (5.50)	2.77 (.61)	.55	.00	.977
NS4.Disorderliness	−.26^**^	7	16.64 (4.59)	.44	16.28 (4.26)	2.32 (.60)	.39	17.11 (4.96)	2.44 (.70)	.49	−.18	.083
**NS. Novelty Seeking**	−.38^**^	**35**	**92.49 (14.94)**	**.74**	**91.50 (14.76)**	**2.61 (.42)**	**.74**	**93.77 (15.13)**	**2.67 (.43)**	**.74**	**−.15**	**.150**
HA1.Anticipatory worry	.05	11	28.54 (6.63)	.64	29.76 (6.98)	2.70 (.63)	.68	26.95 (5.80)	2.45 (.52)	.54	.43	<.000
HA2.Fear of uncertainty	.16^**^	7	25.49 (4.93)	.60	26.82 (4.70)	3.83 (.67)	.58	23.78 (4.70)	3.39 (.67)	.53	.64	<.000
HA3.Shyness with strangers	.10	7	20.80 (5.82)	.72	20.65 (5.89)	2.95 (.84)	.74	20.99 (5.74)	2.99 (.82)	.70	−.05	.584
HA4.Fatigability	.06	8	22.13 (5.60)	.60	22.86 (5.97)	2.85 (.74)	.63	21.18 (4.93)	2.64 (.61)	.52	.30	.004
**HA. Harm Avoidance**	.13^*^	**33**	**96.97 (16.24)**	**.80**	**100.11 (16.48)**	**3.03 (.49)**	**.81**	**92.91 (15.03)**	**2.81 (.45)**	**.77**	**.45**	**<.000**
RD1.Sentimentality	.07	8	29.70 (4.80)	.51	31.01 (4.53)	3.87 (.56)	.52	27.99 (4.61)	3.49 (.57)	.42	.66	<.000
RD2.Opennes to warm	−.13^**^	10	36.04 (7.48)	.75	37.52 (7.12)	3.75 (.71)	.73	34.13 (7.52)	3.41 (.75)	.74	.73	<.000
RD3.Attachment	−.27^**^	6	21.80 (5.42)	.72	22.69 (5.30)	3.78 (.88)	.72	20.66 (5.37)	3.44 (.89)	.72	.38	<.000
RD4.Dependence	−.16^**^	6	20.62 (4.25)	.48	20.81 (4.22)	3.46 (.70)	.46	20.37 (4.28)	3.39 (.71)	.51	.10	.325
**RD. Reward Dependence**	−.18^**^	**30**	**108.17 (15.52)**	**.80**	**112.05 (14.50)**	**3.73 (.48)**	**.78**	**103.16 (15.40)**	**3.43 (.51)**	**.80**	**.59**	**<.000**
PS1.Eagerness of effort	.15^**^	9	31.65 (6.11)	.64	31.60 (6.14)	3.51 (.68)	.66	31.70 (6.10)	3.52 (.67)	.64	−.01	.880
PS2.Work hardened	−.01	8	27.67 (5.71)	.68	27.08 (6.20)	3.38 (.77)	.73	28.43 (4.93)	3.55 (.61)	.55	−.24	.021
PS3.Ambitious	−.09	10	27.92 (6.99)	.71	26.77 (6.89)	2.67 (.68)	.70	29.40 (6.87)	2.94 (.68)	.71	−.38	<.000
PS4.Perfectionist	.07	8	25.58 (5.79)	.63	25.39 (6.01)	3.17 (.75)	.67	25.82 (5.50)	3.22 (.68)	.60	−.07	.483
**PS. Persistence**	**.03**	**35**	**112.82 (19.47)**	**.86**	**110.86 (20.09)**	**3.16 (.57)**	**.87**	**115.36 (18.39)**	**3.29 (.52)**	**.84**	**−.23**	**.028**
SD1.Responsability	−.19^**^	8	31.77 (6.16)	.76	31.65 (6.32)	3.95 (.79)	.76	31.91 (5.95)	3.98 (.74)	.77	−.04	.687
SD2.Purposefulness	−.01	6	23.50 (4.45)	.61	23.15 (4.67)	3.85 (.77)	.63	23.96 (4.10)	3.99 (.68)	.56	−.18	.082
SD3.Resourcefulness	−.19^**^	5	18.83 (3.79)	.56	18.48 (3.80)	3.69 (.76)	.53	19.30 (3.72)	3.86 (.74)	.60	−.21	.041
SD4.Self-acceptance	.06	10	35.09 (8.02)	.76	35.46 (8.16)	3.54 (.81)	.77	34.61 (7.84)	3.46 (.78)	.76	.10	.318
SD5.Enlightened second nature	.09	11	40.71 (6.43)	.64	40.61 (6.65)	3.69 (.60)	.66	40.85 (6.16)	3.71 (.56)	.61	−.03	.721
**SD. Self-directiveness**	**−.04**	**40**	**149.93 (20.21)**	**.85**	**149.37 (21.22)**	**3.73 (.53)**	**.86**	**150.66 (18.87)**	**3.76 (.47)**	**.84**	**−.06**	**.545**
CO1.Social acceptance	−.19^**^	8	32.20 (5.17)	.69	32.46 (5.00)	4.05 (.62)	.67	31.86 (5.38)	3.98 (.67)	.71	.11	.266
CO2.Empathy	−.18^**^	5	18.77 (3.60)	.51	19.42 (3.44)	3.88 (.68)	.50	17.95 (3.64)	3.59 (.72)	.48	.41	.000
CO3.Helpfulness	−.07	8	32.16 (4.02)	.49	32.33 (3.68)	4.04 (.46)	.36	31.93 (4.42)	3.99 (.55)	.61	.1	.350
CO4.Compassion	.05	7	29.14 (4.99)	.77	29.67 (4.68)	4.23 (.66)	.75	28.46 (5.31)	4.06 (.75)	.79	.24	.022
CO5.Pure-hearted conscience	−.02	8	30.36 (4.56)	.33	30.53 (4.23)	3.81 (.52)	.26	30.15 (4.97)	3.76 (.62)	.41	.08	.428
**CO. Cooperativeness**	−.11^*^	**36**	**142.65 (15.22)**	**.80**	**144.43 (13.63)**	**4.01 (.38)**	**.76**	**140.36 (16.84)**	**3.89 (.46)**	**.84**	**.26**	**.013**
ST1.Self-forgetful	.12^*^	10	28.29 (7.31)	.72	28.47 (7.24)	2.84 (.72)	.71	28.05 (7.41)	2.80 (.74)	.73	.05	.579
ST2.Transpersonal identification	.37^**^	8	20.19 (6.21)	.70	20.27 (6.07)	2.53 (.75)	.67	20.10 (6.41)	2.51 (.80)	.73	.02	.795
ST3.Spiritual acceptance	.29^**^	8	18.02 (5.99)	.70	18.57 (6.21)	2.32 (.77)	.71	17.31 (5.62)	2.16 (.70)	.69	.21	.046
**ST. Self-transcendence**	.31^**^	**26**	**66.51 (15.92)**	**.84**	**67.32 (16.12)**	**2.58 (.62)**	**.85**	**65.46 (15.64)**	**2.51 (.60)**	**.84**	**.11**	**.269**

**Notes.**

Correlations:

** *p* < .001.

* *p* < .05.

*** Scalated scores were computed as raw score/number of items to enable comparability across dimensions.

To date, the TCI-R has been adapted in various languages and cross-cultural contexts with clinical and non-clinical samples, including in Italy (Fossati et al., 2007), Belgium (Hansenne, Delhez & Cloninger, 2005), France (Pelissolo et al., 2005), the United States (Cloninger, Przybeck & Svrakic, 1999), Spain (Gutiérrez-Zotes et al., 2004), the Czech Republic (Preiss et al., 2007), Brazil (Goncalves & Cloninger, 2010), Bulgaria (Tilov et al., 2012), Mexico (Fresán et al., 2011) and Serbia (Dzamonja-Ignjatovic et al., 2010). These validation studies have yielded variable results with regard to the psychometric properties of the model. Thus, not all studies obtained similar values of reliability on the scales. The results are inconsistent when the variables of age and sex in each dimension are analyzed. A study based on a random sample is necessary to clarify the bias from non-representativeness by sex and age in other samples.

The majority of the validation studies examining normal populations were conducted with volunteers and/or students, which indicates that there is a self-selection bias. This type of sample selection may not represent the general population in terms of demographic and psychological variables given that it does not consider the moderating effects of these variables. Conclusions generated from studies with these samples make it difficult to extrapolate the results to the general population and may influence the psychometric properties of reliability and construct validity regarding this instrument. The personality dimensions may be influenced by demographic factors, such as age, sex and level of education (Mendlowicz & Girardin, 2000). One study found a positive association between job status and the TCI dimensions of RD, CO and ST (Mendlowicz & Girardin, 2000). Men employed in sectors characterized by outdoor manual work had lower levels of C, whereas men employed in service industries scored higher in ST (Al-Halabí et al., 2010). In that study, other socio-demographic variables also influenced the personality dimensions. Independent of age, NS was higher in women with third-level educations and was markedly lower in women who were homemakers. Men with third-level educations and unmarried men with long-term partners had lower levels of HA. There was an association between education and RD in women such that increasing educational levels were associated with increasing RD. Independent of age, employed men and unmarried men with long-term partners had increased SD. In women, higher educational levels were associated with increased C, whereas women with one sibling had lower C.

Saliba & Ostojic (2014) in a work that examines the influence of personality factors on willingness to participate in studies, suggest that personality factors affect a person’s decision to participate in a study. In a sample of volunteers, the Myers-Briggs Type Inventory-Form M (MBTI-M) was used to assess personality type with the result that a number of personality types were found to be over-represented and under-represented when compared with the United States National Representative Sample (US NRS). However, Goldberg et al. (1998) found that the relationships among demographic and self-reported personality variables were quite small when both sets of variables were assessed in a large and reasonably representative sample of working adults.

To our knowledge, only a few validation studies of the TCI model have been performed with randomly selected populations (Cloninger et al., 1994). This study was conducted with a sample that was representative of the general population and had the following two objectives: (1) to obtain information regarding the sex and age ranges for the dimensions and facets of the TCI-R and TCI-140 and (2) to analyze the reliability of the scales.

## Method

### Subjects

The sample was randomly selected from the electoral roll of a municipality in Tarragona province (Catalonia, Spain) and was stratified for age and sex in the source population. Letters of invitation to participate in the study were sent to a total of 650 people. Willingness to participate was confirmed by phone call. If someone refused or could not be contacted following three different phone calls (at different times on different days), they were replaced by the next available candidate of the same age and sex on the list. Inclusion criteria were the following: Individuals of Caucasian origin with the capability of understanding the nature of the study and who did not have serious diseases that prevented participation. The answer format was self-administered. When any subject was illiterate, the questionnaire was read out by a member of the research team.

There were 367 subjects (207 females and 160 males) who were between 18- and 77-years-old. The average age was 43.09 years (SD = 15.32) for the males and 42.31 years (SD = 15.03) for the females. There were no significant age differences between the males and females according to a *t*-test, which revealed a small effect size (*d* = 0.05). The sample was comprised as follows: 70.57% (*n* = 259) had completed elementary or secondary school, 15.25% (*n* = 56) had completed high school, 11.71% (*n* = 43) had completed a first-level or higher degree and 2.45% (*n* = 9) were illiterate. Regarding civil status, 25.6% (*n* = 94) were single, 64.9% (*n* = 238) were married, 2.7% (*n* = 10) lived with a partner, 2.7% (*n* = 10) were widowed and 3.6% (*n* = 13) were separated. There were two cases missing for the civil status results. All procedures were conducted in accordance with the Declaration of Helsinki. Ethical approval was obtained from the Institutional Review Board, and all subjects provided written informed consent.

### Instrument

Temperament and Character Inventory—Revised (TCI-R) and TCI-140. The TCI-R is a 240-item self-administered questionnaire designed to measure four dimensions of temperament and three dimensions of character as shown in Table 1. We used the Spanish version of the TCI-R, described in Gutiérrez-Zotes et al. (2004). The response option format of the TCI-R ranged from 1 = *definitely false* to 5 = *definitely true*. Regarding the abbreviated TCI-R inventory, the TCI-140 consists of the first 140 items from the TCI-R. Of these 140 items, 136 items relate to the seven temperament and character domains and the remaining four items measure response accuracy/carelessness. An abbreviated form of the TCI-R is required when the application time is limited, for example when it is necessary to measure the personality along with other variables in the context of research or diagnosis.

### Statistical analyses

Descriptive statistics, mean differences with *t*-tests, and alpha internal consistencies for all of the subjects were analyzed according to sex. Effect sizes were estimated from the *t*-tests with Cohen’s *d*. Univariate analyses of variance followed by Bonferroni tests were used to compare the TCI-R dimension scores according to age group (cohorts 18–35 years, 36–50 years and 51–77 years) and sex. Inter-correlations among the seven dimensions of the TCI-R were obtained with Pearson statistical tests. All of the analyses were performed with version 17.0 of the SPSS (Chicago, IL) statistical software.

## Results

### Descriptive analyses, age correlations, and reliability

Administering the TCI-R and TCI-140 to a general sample revealed that kurtosis and skewness were almost zero for the seven principal dimensions with the exception of Cooperativeness (C). The skewness results for C for the TCI-R and TCI-140 were −.84 and −.84, respectively, whereas the kurtosis results were 1.62 and 1.53, respectively. Given the mild non-normality of Cooperativeness, non-parametric equivalents were tested, which did not change the current results (not shown). Tables 1 and 2 present the descriptive data and the *α* reliability for the whole sample and according to sex.

**Table 2:** TCI-140 descriptive, alpha, means comparison for sex and, correlations with age.

Facets and scales	*r* Age	Items	All (*n* = 367)		Females (*n* = 207)			Males (*n* = 160)				
			M (SD)	*α*	M (SD)	M (SD)^***^	*α*	M (SD)	M (SD)^***^	*α*	Cohen’s *d*	*p*
NS1.Exploratory exctability	−.17^**^	5	13.63 (3.32)	.15	13.53 (3.34)	2.70 (.66)	.17	13.76 (3.29)	2.75 (.65)	.16	−.06	.498
NS2.Impulsiveness	.02	5	13.23 (3.72)	.44	13.09 (3.78)	2.61 (.75)	.47	13.41 (3.65)	2.68 (.73)	.41	−.08	.414
NS3.Extravagance	−.24^**^	5	12.43 (3.85)	.56	12.41 (4.01)	2.48 (.80)	.63	12.46 (3.65)	2.49 (.73)	.48	−.01	.908
NS4.Disorderliness	−.25^**^	5	11.56 (3.59)	.35	11.31 (3.24)	2.26 (.64)	.25	11.89 (3.98)	2.37 (.79)	.44	−.15	.135
**NS. Novelty Seeking**	−.24^**^	**20**	**50.86 (9.46)**	**.63**	**50.35 (9.31)**	**2.51 (.46)**	**.63**	**51.53 (9.64)**	**2.57 (.48)**	**.63**	**−.12**	**.235**
HA1.Anticipatory worry	.05	5	14.50 (3.82)	.43	15.30 (3.89)	3.06 (.77)	.48	13.46 (3.47)	2.69 (.69)	.29	.49	<.000
HA2.Fear of uncertainty	.08	5	17.15 (4.19)	.62	18.22 (4.10)	3.64 (.82)	.63	15.76 (3.90)	3.15 (.78)	.52	.61	<.000
HA3.Shyness with strangers	.05	5	14.45 (4.56)	.67	14.22 (4.63)	2.84 (.92)	.70	14.75 (4.45)	2.95 (.89)	.64	−.09	.267
HA4.Fatigability	−.04	5	14.20 (3.85)	.49	14.47 (4.08)	2.89 (.81)	.52	13.84 (3.51)	2.76 (.70)	.42	.16	.111
**HA. Harm Avoidance**	**.05**	**20**	**60.31 (11.52)**	**.76**	**62.23 (11.56)**	**3.11 (.57)**	**.76**	**57.83 (11.02)**	**2.89 (.55)**	**.74**	**.38**	**<.000**
RD1.Sentimentality	.10^*^	5	18.11 (3.69)	.45	19.30 (3.43)	3.86 (.68)	.47	16.56 (3.43)	3.31 (.68)	.27	.79	<.000
RD2.Opennes to warm	−.10^*^	5	17.31 (4.43)	.63	17.99 (4.34)	3.59 (.86)	.62	16.43 (4.40)	3.28 (.88)	.62	.35	.001
RD3.Attachment	−.23^**^	5	18.87 (4.49)	.68	19.52 (4.29)	3.90 (.85)	.66	18.03 (4.61)	3.60 (.92)	.69	.33	.002
RD4.Dependence	−.19^**^	5	16.85 (3.82)	.47	17.13 (3.75)	3.42 (.75)	.43	16.48 (3.89)	3.29 (.77)	.52	.17	.107
**RD. Reward Dependence**	−.17^**^	**20**	**71.15 (10.80)**	**.72**	**73.95 (9.97)**	**3.69 (.49)**	**.68**	**67.52 (10.79)**	**3.37 (.53)**	**.72**	**.62**	**<.000**
PS1.Eagerness of effort	.15^**^	5	17.40 (4.21)	.59	17.24 (4.17)	3.44 (.83)	.59	17.60 (4.27)	3.52 (.85)	.58	−.08	.412
PS2.Work hardened	.02	5	17.42 (3.92)	.57	16.97 (4.11)	3.39 (.82)	.61	18.01 (3.59)	3.60 (.71)	.48	−.26	.012
PS3.Ambitious	−.04	5	13.44 (3.68)	.55	12.85 (3.61)	2.57 (.72)	.56	14.20 (3.65)	2.84 (.73)	.51	−.37	<.000
PS4.Perfectionist	.09	5	15.61 (4.35)	.65	15.36 (4.40)	3.07 (.88)	.67	15.93 (4.27)	3.18 (.85)	.64	−.13	.215
**PS. Persistence**	**.08**	**20**	**63.87 (12.28)**	**.81**	**62.43 (12.42)**	**3.12 (.62)**	**.82**	**65.75 (11.88)**	**3.28 (.59)**	**.79**	**−.27**	**.010**
SD1.Responsability	−.20^**^	5	19.37 (4.24)	.67	19.30 (4.27)	3.86 (.85)	.65	19.46 (4.22)	3.89 (.84)	.69	−.03	.724
SD2.Purposefulness	−.07	5	20.13 (3.92)	.61	19.75 (4.11)	3.95 (.82)	.63	20.63 (3.60)	4.12 (.72)	.57	−.22	.033
SD3.Resourcefulness	−.14^**^	3	11.95 (2.68)	.59	11.77 (2.76)	3.92 (.92)	.60	12.18 (2.56)	4.06 (.85)	.58	−.15	.147
SD4.Self-acceptance	.10^*^	2	7.93 (2.12)	.56	8.15 (1.97)	4.07 (.98)	.42	7.65 (2.28)	3.82 (1.14)	.69	.23	.029
SD5.Enlightened second nature	−.00	5	17.45 (4.08)	.55	17.21 (4.22)	3.44 (.84)	.56	17.76 (3.89)	3.55 (.77)	.52	−.13	.196
**SD. Self-directiveness**	−.11^*^	**20**	**76.85 (12.29)**	**.82**	**76.20 (12.76)**	**3.81 (.63)**	**.83**	**77.70 (11.64)**	**3.88 (.58)**	**.81**	**−.12**	**.246**
CO1.Social acceptance	−.19^**^	4	16.40 (3.29)	.65	16.53 (3.12)	4.13 (.78)	.60	16.23 (3.49)	4.05 (.87)	.69	.09	.390
CO2.Empathy	−.20^**^	4	14.54 (3.11)	.42	15.07 (3.02)	3.76 (.75)	.42	13.86 (3.11)	3.46 (.77)	.38	.39	<.000
CO3.Helpfulness	−.00	4	15.77 (2.33)	.22	15.87 (2.19)	3.96 (.54)	.04	15.64 (2.50)	3.91 (.62)	.39	.09	.349
CO4.Compassion	.01	4	17.51 (3.28)	.77	17.85 (3.14)	4.46 (.78)	.76	17.07 (3.41)	4.26 (.85)	.77	.23	.024
CO5.Pure-hearted conscience	−.09	4	16.36 (2.83)	.34	16.74 (2.53)	4.18 (.63)	.29	15.88 (3.12)	3.97 (.78)	.37	.30	.005
**CO. Cooperativeness**	−.15^**^	**20**	**80.60 (9.67)**	**.74**	**82.08 (8.66)**	**4.10 (.43)**	**.69**	**78.70 (10.57)**	**3.93 (.52)**	**.78**	**.34**	**.001**
ST1.Self-forgetful	.13^**^	6	16.19 (4.89)	.62	16.35 (4.82)	2.72 (.80)	.60	15.99 (4.97)	2.66 (.82)	.65	.07	.579
ST2.Transpersonal identification	.36^**^	5	12.27 (4.29)	.64	12.49 (4.37)	2.49 (.87)	.65	11.99 (4.17)	2.39 (.83)	.63	.11	.265
ST3.Spiritual acceptance	.32^**^	5	9.49 (4.26)	.69	9.84 (4.55)	1.96 (.91)	.71	9.03 (3.81)	1.80 (.76)	.64	.19	.067
**ST. Self-transcendence**	.32^**^	**16**	**37.96 (11.22)**	**.82**	**38.69 (11.47)**	**2.41 (.71)**	**.83**	**37.02 (10.84)**	**2.31 (.67)**	**.82**	**.14**	**.269**

**Notes.**

Correlations:

** *p* < .001.

* *p* < .05.

*** Scalated scores were computed as raw score/number of items to enable comparability across dimensions.

On the TCI-R, women scored higher in HA (*d* = .45), RD (*d* = .59) and C (*d* = .26), whereas men scored higher in PS (*d* = − .23). These differences were also evident for the TCI-140, with higher scores for women in HA (*d* = .38), RD (*d* = .62) and C (*d* = .34), whereas men scored higher in PS (*d* = − .27). The Cohen ‘*d*’ values revealed medium effect sizes.

Several dimensions had negative correlations with age, including NS (NS1, NS3 and NS4), RD (RD2, RD3 and RD4), SD1, SD2 and C (C1 and C2), whereas positive coefficients were obtained for HA (HA2), PS1, ST (ST1, ST2 and ST3). On the TCI-140, negative coefficients with age were evident for NS (NS1, NS3 and NS4), RD (RD2, RD3 and RD4), SD (SD1, SD3 and SD4), C (C1 and C2), whereas positive coefficients were evident for RD1, PS1 and ST (ST1, ST2 and ST3). The alphas were between 0.74 and 0.87 for the TCI-R and between 0.63 and 0.83 for the TCI-140.

Table 3 presents the correlation coefficients for the TCI-R and TCI-140 scales (in brackets). NS correlated positively with RD (TCI-R *r* = .19 and TCI-140 *r* = 0.12) and ST (TCI-140 *r* = .20) and correlated negatively with SD (TCI-R *r* = − .15 and TCI-140 *r* = − .21) and C (TCI-140 *r* = − .11). HA correlated negatively with NS (TCI-R *r* = − .33 and TCI-140 *r* = − .25), RD (TCI-R *r* = − .15), PS (TCI-R *r* = − .36 and TCI-140 *r* = − .28), SD (TCI-R *r* = − .41 and TCI-140 *r* = − .38) and C (TCI-R *r* = − .27 and TCI-140 *r* = − .15). RD correlated positively with PS (TCI-R *r* = .14), SD (TCI-R *r* = .23 and TCI-140 *r* = .22), C (TCI-R *r* = .52 and TCI-140 *r* = .42) and ST (TCI-R *r* = .11). PS correlated positively with ST (TCI-R *r* = 0.35 and TCI-140 *r* = .32), and SD correlated positively with C (TCI-R *r* = .51 and TCI-140 *r* = 0.44) and correlated negatively with ST (TCI-R *r* = − .28 and TCI-140 *r* = − .38).

**Table 3:** Correlations among Temperament and Character scales of TCI-R and TCI-140.

	NS	HA	RD	PS	SD	C	ST
NS	–						
HA	−.33^**^ (−.25^**^)	–					
RD	.19^**^ (.12^*^)	−.15^**^ (−.07)	–				
PS	−.05 (−.00)	−.36^**^ (−.28^**^)	.14^**^ (.09)	–			
SD	−.15^**^ (−.21^**^)	−.41^**^ (−.38^**^)	.23^**^ (.22^**^)	.09 (.06)	–		
C	−.02 (−.11^*^)	−.27^**^ (−.15^**^)	.52^**^ (.42^**^)	.04 (−.05)	.51^**^ (.44^**^)	–	
ST	.05 (.20^**^)	−.06 (−.08)	.11^*^ (.02)	.35^**^ (.32^**^)	−.28^**^ (−.38^**^)	−.01 (−.08)	–

**Notes.**

Correlations:

** *p* < .001.

* *p* < .05.

NS Novelty Seeking HA Harm Avoidance RD Reward Dependence PS Persistence SD Self-directedness CO Cooperativeness ST Self-transcendence TCI-140 correlations in brackets

The mean scores and standard deviations for the groups according to age and sex are presented in Table 4 for the TCI-R and Table 5 for the TCI-140. For the TCI-R, differences were evident among the age cohorts for the temperament dimensions of NS, HA and RD, whereas, for the TCI-140, these differences were only evident for NS and RD. On the TCI-R, young subjects scored higher in NS than age group 2 (*p* < .000) and age group 3 (*p* < .000). In contrast, subjects between the ages of 51 and 77 years scored significantly higher in HA than those between 18 and 35 years (*p* = .010). Younger subjects also scored higher in RD than those in group 3 (*p* < .000). The results for the TCI-140 were similar, with higher scores in NS for young people compared with group 2 (*p* = .012) and group 3 (*p* < .000) and the youngest subjects scored higher in RD than those in group 3 (*p* = .001). The ST character dimensions showed similar increases for both the TCI-R and TCI-140, with differences between groups 1 and 3 and groups 2 and 3 (*p* < .000). On the TCI-140, SD and C decreased with age, with differences between the youngest and oldest subjects at *p* = .041 and *p* = .025, respectively.

**Table 4:** Means scores of the TCI-R dimensions by age groups and sex.

Dimension	Age cohorts			
	18–35 (*n* = 149)	36–50 (*n* = 96)	51–77 (*n* = 122)	Statistics
NS All	98.75 (14.14)	91.41 (13.94)	85.70 (13.53)	Age: *F* = 29, *p* < .000
Men	99.60 (15.53)	93.50 (12.56)	87.30 (14.22)	Sex: *F* = 3.4, *p* = .064
Women	98.15 (13.14)	89.50 (14.96)	84.47 (12.95)	Age × sex: *p* = .779
HA All	93.91 (16.43)	98.39 (15.53)	99.59 (16.07)	Age: *F* = 5.6, *p* = .004
Men	88.47 (15.09)	96.23 (13.17)	95.15 (15.48)	Sex: *F* = 17.7, *p* < .000
Women	97.69 (16.34)	100.38 (17.31)	103 (15.78)	Age × sex: *p* = .463
RD All	110.79 (14.86)	109.47 (16)	103.95 (15.15)	Age: *F* = 6.7, *p* = .001
Men	104.31 (15.48)	104.47 (14.07)	100.71 (16.38)	Sex: *F* = 31.4, *p* < .000
Women	115.29 (12.67)	114.08 (16.41)	106.44 (13.74)	Age × sex: *p* = .336
PS All	112.12 (20.25)	113.29 (19.43)	113.31 (18.65)	Age: *F* = .1, *p* = .853
Men	115.09 (20)	111.91 (17.13)	118.66 (17.23)	Sex: *F* = 3.6, *p* = .057
Women	110.06 (20.27)	114.56 (21.42)	109.20 (18.77)	Age × sex: *p* = .073
SD All	151.07 (20.39)	149.73 (19.58)	148.69 (20.56)	Age: *F* = .4, *p* = .611
Men	151.75 (19.25)	151.45 (18.19)	148.71 (19.22)	Sex: *F* = .4, *p* = .492
Women	150.60 (21.25)	148.16 (20.83)	148.68 (21.68)	Age × sex: *p* = .840
C All	143.71 (14.17)	144.41 (14.98)	139.99 (16.38)	Age: *F* = 3.0, *p* = .048
Men	141.06 (17.08)	143.23 (14.51)	137.07 (18.14)	Sex: *F* = 6.0, *p* = .014
Women	145.54 (11.48)	145.50 (15.47)	142.23 (14.64)	Age × sex: *p* = .768
ST All	61.97 (15.62)	65.13 (14.57)	73.14 (15.16)	Age: *F* = 19.2, *p* < .000
Men	59.57 (15.45)	64.63 (13.50)	72.98 (14.69)	Sex: *F* = 1.1, *p* = .276
Women	63.63 (15.61)	65.60 (15.61)	73.27 (15.62)	Age × sex: *p* = .563

**Notes.**

NS Novelty Seeking HA Harm Avoidance RD Reward Dependence PS Persistence SD Self-directedness CO Cooperativeness ST Self-transcendence

**Table 5:** Means scores of the TCI-140 dimensions by age groups and sex.

Dimension	Age cohorts			
	18–35 (*n* = 149)	36–50 (*n* = 96)	51–77 (*n* = 122)	Statistics
NS All	53.51 (9.82)	50.03 (9.22)	48.29 (8.38)	Age: *F* = 10.3, *p* < .000
Men	53.55 (10.87)	50.43 (8.99)	50.16 (8.38)	Sex: *F* = 1.9, *p* = .160
Women	53.48 (9.09)	49.66 (9.51)	46.85 (8.15)	Age × sex: *p* = .341
HA All	58.97 (11.76)	61.25 (11.06)	61.21 (11.51)	Age: *F* = 2.2, *p* = .103
Men	55.50 (11.29)	60.02 (10.34)	58.62 (10.96)	Sex: *F* = 12.4, *p* < .000
Women	61.38 (11.54)	62.38 (11.67)	63.20 (11.61)	Age × sex: *p* = .498
RD All	72.85 (10.45)	72.04 (11.14)	68.36 (10.50)	Age: *F* = 6.1, *p* = .002
Men	68.27 (11.04)	68.45 (9.92)	65.84 (11.23)	Sex: *F* = 34.2, *p* < .000
Women	76.03 (8.76)	75.34 (11.26)	70.30 (9.53)	Age × sex: *p* = .410
PS All	62.79 (12.73)	64.13 (11.87)	64.99 (12.02)	Age: *F* = 1.2, *p* = .297
Men	64.40 (13.09)	64.41 (10.13)	68.45 (11.58)	Sex: *F* = 5.7, *p* = .017
Women	61.68 (12.42)	63.88 (13.38)	62.33 (11.76)	Age × sex: *p* = .233
SD All	78.42 (12.19)	77.13 (11.90)	74.72 (12.49)	Age: *F* = 3.1, *p* = .045
Men	79.45 (11.53)	77.91 (11.67)	75.50 (11.59)	Sex: *F* = 1.3, *p* = .239
Women	77.71 (12.65)	76.42 (12.19)	74.11 (13.19)	Age × sex: *p* = .993
C All	81.77 (8.78)	81.20 (9.54)	78.70 (10.57)	Age: *F* = 3.8, *p* = .022
Men	79.78 (10.38)	80.15 (9.19)	76.18 (11.62)	Sex: *F* = 10.4, *p* = .001
Women	83.15 (7.24)	82.18 (9.85)	80.63 (9.31)	Age × sex: *p* = .649
ST All	34.59 (10.45)	36.98 (10.52)	42.84 (11.01)	Age: *F* = 21.6, *p* < .000
Men	32.95 (9.70)	36.19 (9.90)	43 (12.19)	Sex: *F* = 2.1, *p* = .142
Women	35.73 (10.85)	37.72 (11.11)	43.15 (11.26)	Sex: *F* = 1.6, *p* = .730

**Notes.**

NS Novelty Seeking HA Harm Avoidance RD Reward Dependence PS Persistence SD Self-directedness CO Cooperativeness ST Self-transcendence

Thus, age and sex had independent effects on personality differences, with no significant results with regard to interactions for either variable.

## Discussion

The present study examined differences in personality dimensions according to age and sex, as well as the reliability of the TCI-R and its abbreviated version, the TCI-140, in a representative sample of the general population. Given its method of sample selection, this study addresses a number of the most common problems related to questionnaire validation and selection bias. Selection bias implies that the subjects analyzed in a study differ from non-participants, for example, the volunteers in research studies may have a different personality characteristics compared to the general population (Dollinger & Leong, 1993; Marcus & Schütz, 2005; Saliba & Ostojic, 2014). In a study utilizing the five-factor model of personality, Dollinger & Leong (1993) found that volunteers were more agreeable, more open to experience, and somewhat more extroverted. Marcus & Schütz (2005) reported higher levels of extroversion, openness to experience, and narcissism in volunteers compared to non-volunteers in a study examining both self- and observer ratings. They noted that non-response biases may have significant implications for representativeness when studying volunteers using surveys and personality test norms. Another study found relationships among educational level and a number of properties of NEO, including a positive correlation with Openness (*r* = 0.37) and a negative correlation with Conscientiousness (−0.22) (Vassend & Skrondal, 1995).

In the present study NS scores were lower than in others. In a Belgian version of the Inventory, Hansenne, Delhez & Cloninger (2005) found scores approaching 100 in NS when the questionnaire was administered to psychology students and their relatives. Similar NS scores have also been reported with different types of study volunteers in Brazil (Goncalves & Cloninger, 2010), Mexico (Fresán et al., 2011), Bulgaria (Tilov et al., 2012), Serbia (Dzamonja-Ignjatovic et al., 2010) and the USA (Cloninger, Przybeck & Svrakic, 1999). Lower scores than those found in the current study for RD, SD and C were evident in a Czech version of the inventory. A higher score on RD in our country can be derived from the extrovert and social personality of Mediterranean society. Similarly, versions administered in the USA (Cloninger, Przybeck & Svrakic, 1999) and Serbia (Dzamonja-Ignjatovic et al., 2010) revealed higher ST scores than those found with the current sample.

The reliability of the TCI-R, as measured by the internal consistency between items of the same dimension, showed values of 0.74 (NS in women) to 0.87, which are values considered to be in the ‘good-to-excellent’ range. In general, these reliability values are similar to or a little lower than those obtained in non-patient cohorts (Cloninger, Przybeck & Svrakic, 1999; Hansenne, Delhez & Cloninger, 2005; Farmer & Goldberg, 2008; Preiss et al., 2007; Goncalves & Cloninger, 2010; Tilov et al., 2012; Dzamonja-Ignjatovic et al., 2010) and in patient cohorts (Fossati et al., 2007; Pelissolo et al., 2005). Previous studies with volunteer subjects (Hansenne, Delhez & Cloninger, 2005; Gutiérrez-Zotes et al., 2004) yielded higher values for some of the dimensions, including HA and RD. However, Fresán et al. (2011) reported similar scores to ours for RD. For the TCI-140, the reliability varied from 0.63 (NS) and 0.72 (RD) to 0.82 (SD). These reliability coefficients are inferior to those obtained in samples in the USA (Farmer & Goldberg, 2008). For the TCI-140, NS had a reliability of 0.64 in an Israeli version, which was administered to volunteers (Zohar & Cloninger, 2011). NS was higher (0.69) in a Spanish version administered to psychiatric patients (Gutiérrez-Zotes et al., 2005). Despite the lower reliability, an abbreviated instrument of TCI-R may be a useful instrument in order to assess Cloninger’s model of the 7 personality dimensions when the time to respond needs to be reduced.

These differences in reliability between our results with a randomly selected sample and those from previous research with volunteers may be explained by a cognitive-attentional factor associated with the type of subject being examined. For example, university students and volunteers are interested and highly motivated in participating in studies and have better cognitive and attentional capacities that may improve their consistency of responses. The use of volunteer student subjects in highly homogeneous contexts, such as universities, may affect the external validity of a study given that the subjects share attributes, dispositions and environments with regard to their chosen specializations.

Several of the facets did not obtain acceptable alphas. This has been reported before for other TCI-R versions and samples (Fossati et al., 2007; Hansenne, Delhez & Cloninger, 2005; Preiss et al., 2007; Goncalves & Cloninger, 2010; Tilov et al., 2012; Dzamonja-Ignjatovic et al., 2010; Snopek et al., 2012; Martinotti et al., 2008). However, unreliable scales do not coincide between studies, suggesting that this result is probably due to the small number of items together with particularities of each sample. Future studies should test subscales for reliability in their own samples before starting other analyses.

Comparisons according to sex revealed that men had significantly lower scores in HA, RD and C than women, which is consistent with previous research (Hansenne, Delhez & Cloninger, 2005; Goncalves & Cloninger, 2010; Aluja et al., 2010). Men scored significantly higher in PS (PS2 and PS3), which is also consistent with previous research (Hansenne, Delhez & Cloninger, 2005; Goncalves & Cloninger, 2010). Pelissolo et al. (2005) found that men had higher scores in RD, C and SD but not in HA, whereas Preiss et al. (2007) only found differences in women, with higher scores in RD and C. Women scoring significantly higher than men has been consistently reported by researchers studying normative and clinical samples in North America, Europe, and Japan (Mendlowicz & Girardin, 2000). This association might reflect sex-specific in noradrenergic systems. Mendlowicz & Girardin (2000) hypothesize that increased RD (sentimental and socially sensitive) may be one of the mechanisms that maximize women’s parenting effectiveness. In a meta-analysis, women scored higher than men in HA in almost all studies (Miettunen et al.). An explanation could be the relation of HA with anxiety which are more common among women (Cloninger, Svrakic & Przybeck, 2006; Cloninger, Bayon & Svrakic, 1998).

The age of the subjects was negatively associated with NS, RD and C and positively associated with HA and ST. Pelissolo et al. (2005) found positive correlations with ST, SD and C and a negative correlation with NS. Hansenne, Delhez & Cloninger (2005) found a negative correlation with NS and a positive correlation with SD. Young subjects between 18 and 35 years had higher scores than older subjects in NS and RD. Similar scores were reported by Fresán et al. (2011). Subjects between 51 and 77 years scored higher in both HA and ST. A higher score in HA in the older group is consistent with previous research (Preiss et al., 2007; Fresán et al., 2011; Aluja et al., 2010).

In sum, this study shows that the psychometric properties of the Temperament and Character scales are suitable for the TCI-R but that there is a loss of internal consistency in the NS dimension of the TCI-140. The youngest subjects scored higher in Novelty Seeking and Reward Dependence, whereas the older subjects scored higher in Harm Avoidance and Self-Transcendence. These new normative data replaces the previously published by our group with volunteers (Gutiérrez-Zotes et al., 2004).

The greatest strength of the current study is the randomness of the sample. However, one possible limitation is the small sample size when the data are analyzed for each sex and age group. Comparisons in the dimensions of the TCI-R by age and sex are made with other studies with volunteers who have a higher socioeconomic status in relation to the subjects of our sample. This demographic difference can limit the comparisons.

Tables with the T scores for each raw score of TCI-R and TCI-140 dimensions can be sent by the authors on request.

## Supplemental Information

10.7717/peerj.1481/supp-1 Supplemental Information 1 TCI-R/TCI-140 raw data

## Additional Information and Declarations

### Data Availability

Raw data is available in the Supplemental Information.
